# Experimental study on changes in metabolic mechanism of papillary thyroid carcinoma complicated with Hashimoto’s thyroiditis

**DOI:** 10.1016/j.heliyon.2023.e20661

**Published:** 2023-10-05

**Authors:** Danyang Sun, Yujie Zhang, Dan Wang, Xue Zhao, Rui Han, Ning Li, Xue Li, Tingwei Li, Peng Wang, Qiang Jia, Jian Tan, Wei Zheng, Lili Song, Zhaowei Meng

**Affiliations:** a Department of Nuclear Medicine, Tianjin Medical University General Hospital Airport Site, Tianjin, China; b Department of Nuclear Medicine, Tianjin Medical University General Hospital, Tianjin, China; c Department of Pathology, Tianjin Medical University General Hospital, Tianjin, China; d Department of Pathology, Tianjin First Central Hospital, Tianjin, China; e School of Traditional Chinese Materia Medica, Tianjin University of Traditional Chinese Medicine, Tianjin, China; f Tianjin Shangmei Cosmetics Co., Ltd, Tianjin, China

**Keywords:** Metabolomic, Hashimoto's thyroiditis (HT), Papillary thyroid cancer (PTC), Metabolism pathway, Biomarkers

## Abstract

**Background:**

Whether the mechanism of thyroid papillary carcinoma (PTC) is the same in patients with a Hashimoto's thyroiditis (HT) background as compared with patients with a normal background remains a highly debated and controversial issue. In this study, we aimed to analyze the differences and similarities of the metabolic mechanism of PTC in normal and HT background, and to explore the relationship between HT and PTC.

**Methods:**

The ultra performance liquid chromatography-quadrupole-time of flight-mass spectrometry (UPLC-Q-TOF/MS) technology was used to analyze 61 PTC patient tissues (31 HT background and 30 normal tissue (NC) background). Potential biomarkers were screened from principal component analysis (PCA) to orthogonal partial least square (OPLS) discriminant analysis. HMDB was searched to identify potential differential metabolites and final metabolic pathway analysis was performed by MetaboAnalyst 5.0. We analyzed the differential metabolites diagnostic accuracy through receiver operating characteristic (ROC) curves analysis.

**Results:**

Seven different metabolites were screened from HT group and NC group, including arginine, glutamic acid, cysteine, citric acid, malic acid, uracil and taurine. Logistic regression model combined with ROC analysis of these 7 biomarkers had good discriminability for PTC (area under operating characteristic curve of HT group and NC group were 0.867 and 0.973, respectively). The HT group had specific metabolic pathways, including aminoacyl-tRNA biosynthesis, glycine, serine and threonine metabolism.

**Conclusions:**

The metabolic profiles of the NC and HT groups had important similarities and differences in PTC. The correlation of PTC with HT may be related to aminoacyl-tRNA biosynthesis, serine and threonine metabolism.

## 1 Introduction

Thyroid cancer is the most common type of endocrine tumor, while papillary thyroid cancer (PTC) accounts for 95% of all cases [1,2]. Hashimoto thyroiditis (HT), an autoimmune disorder, is characterized by lymphocytic infiltration intermingled with the epithelial (follicular) cells normally resident in the thyroid parenchyma [3]. In some PTC patients, lymphocyte infiltration could be found in the postoperative pathology (HT background). Their coexistence has been reported to range from 10% to 58%, arousing great concern [4,5]. Some studies have shown that HT is associated with a significantly increased risk of PTC, but coexistent HT has been reported to be significantly associated with the less aggressive clinicopathologic characteristics of PTC [6]. Other studies have shown no connection between the presence of HT and PTC [[7], [8], [9]]. Whether the mechanism of PTC is the same in patients with an HT background compared with patients with a normal background remains a highly debated and controversial issue.

The similarities in immunology [10], endocrinology [11] and genetics [12], suggest that there is a certain correlation between PTC and HT, however, the specific mechanism between them still needs to be further explored. Metabolomics, as a newly emerging technology, has been used in the study of disease mechanisms and metabolic changes by discovering potential biomarkers and associated metabolic pathways [[13], [14], [15]]. For thyroid diseases, a limited number of previous studies have shown that it could help distinguishing benign thyroid nodules from malignant ones, and detecting thyroid cancer tumor markers [16,17]. Metabolomic profiles are sensitive enough to delineate differences between PTC tissues versus adjacent non-tumor tissues [15,18]. However, thyroid metabolism is complex, and from our literature retrieval, no papers have explored the metabolic mechanisms of thyroid cancer in normal and HT background to explore their relationship.

Therefore, by using the ultra performance liquid chromatography-quadrupole-time of flight-mass spectrometry (UPLC-Q-TOF/MS) method, we intended to analyze the metabolic differences and similarities of thyroid cancer in HT as well as in normal tissue backgrounds to find relevant pathways and explore potential mechanisms, which may provide a new angle and direction for understanding the disease.

## 2 Materials and methods

### 2.1 Clinical sample and sample preparation

Between January 2018 and January 2019, a total of 61 samples were collected from the Department of General Surgery, General Hospital of Tianjin Medical University. Since the clinical characteristics of patients may affect the results of the study, the propensity score matching method (PSM) was used for case-control matching of the data. Our present study included 14 men and 47 women (age range, 23–70 years). With a maximum tumor diameter of 0.2–4.0 cm. Among them, there were 31 cases of PTC tissue samples and paracancerous tissue samples with HT background, and 30 cases of PTC with paracancerous tissue in normal background. We defined them as HT group and NC group, respectively. All patients had undergone surgical thyroidectomy, then fresh thyroid tumor tissues and corresponding normal thyroid tissues were washed with phosphate-buffered saline following and snap-frozen in liquid nitrogen and were subsequently stored at −80 °C until analysis. Histological assessment was conducted based on established criteria of the World Health Organization. Pathological diagnoses were confirmed independently by two pathologists. The clinical stage was classified according to the TNM criteria in US thyroid cancer guidelines [19].

### 2.2 Conditions of UPLC-Q-TOF/MS

Protocol to process the experimental samples was conducted referring to our previous research and experimental methods with modifications [20,21]. Blood stains on the surface of samples were rinsed with normal saline, blot dry with filter paper, and weigh. For each 50 mg of sample, we added 250 μL of methanol/water (4:1, v/v) and homogenized the mixture. The mixtures were placed at −20 °C for 20 min to extract metabolites, and then centrifuged at 12,000 rpm for 10 min at 4 °C and the supernatant was collected. The supernatant was transferred into autosampler vials for UPLC-Q-TOF-MS analysis. Quality control (QC) samples were analyzed at the beginning of the run and injected once after every 16 injections of the randomly sequenced samples, verifying the repeatability and reliability of the UPLC-Q-TOF-MS system. UPLC-Q-TOF/MS analysis was performed within 24 h to ensure the stability of instrument performance and to avoid derivatives.

### 2.3 Screening potential biomarkers

Before analysis, data were normalized by dividing the spectral intensity of each metabolite by the sum of all the metabolites in that spectrum. The preprocessed data were imported into SIMCA 14.0 software (Umetrics, Sweden) to obtain principal component analysis (PCA) scores, orthogonal partial least square discriminant analysis (OPLS-DA) scores, and S-Plot to analyze the overall differences in metabolite distribution between the groups. PCA was performed first to identify possible outliers. Then we use the scale to select the Par data processing mode, complete the automatic fitting, and check for anomalies. If there are anomalies, we delete the corresponding data from the synthesis analysis and check the fit again. When removing outliers, the number of PCA repeats was no more than 3 times. If the outliers increased with the progress of PCA, the group with the least outliers would be usually selected. After the fewest outlier points remaining, the model type selects OPLS-DA to construct the OPLS-DA model and randomizes the order of samples in the dataset. Screening of differential metabolites was mainly based on variable importance in the projection (VIP), univariate test analysis *t*-test, and fold change (FC). If the variable satisfied both VIP>1, *P* < 0.05, and FC > 1.2 (indicating the content of PTC cancer tissue was higher than adjacent tissue) or FC < 0.83 (indicating the content of PTC cancer tissue was lower than adjacent tissue), the variable was considered as a differential metabolite. The OPLS-DA models were sevenfold cross-validated and the quality was evaluated by the R^2^X and Q^2^Y values. For the differential variables obtained from the screening, the molecular weights and molecular formulas of the differential metabolites were initially determined using the element component analysis module in MassLynx software. The human metabolome database (HMDB) was searched for molecular formulas, molar mass, and other information, combined with relevant literature to find potential differential metabolites. These metabolites were introduced into Metaboanalyst 5.0 for analyzing their related metabolic pathways. The metabolic pathways with Raw *P* < 0.05 and Pathway Impact>0.1 were retained.

### 2.4 Statistical analysis

The statistical analysis was carried out using Statistical Program for Social Sciences (SPSS) software, version 22.0 (IBM, Armonk, New York, USA). *t*-test and analysis of variance were used to evaluate differences between continuous variables. Data with a non-normal distribution were tested using the Mann–Whitney *U* test. Chi square-test was used to evaluate differences between categorical variables. The area under the curve (AUC) was analyzed by receiver operating characteristic (ROC) curves to compare the predictive power of significant metabolites between groups. Graphs for descriptive analysis were depicted using GraphPad Prism 8.0 (San Diego, CA, United States) statistical software. P < 0.05 was considered statistically significant.

## 3 Results

### 3.1 Clinical characteristics

The main clinical characteristics of the participants were presented in Table 1. There were no significant differences in age, gender, extra-thyroid extension, tumor multifocality, tumor stage or lymph node metastasis between the two groups (*P* > 0.05).

**Table 1:** Clinical characteristics of the subjects.

Parameter	NC（n = 31）	HT (n = 30)
Age (years)	45.93 ± 12.44	43.62 ± 12.98
Gender		
Male	8 (25.81%)	6（20.00%）
Female	23 (74.19%）	24（80.00%）
Multifocal		
Yes	15（48.39%）	6（20.00%）a
No	16（51.61%）	24（41.38%）
Extrathyroid extension		
positive	6（19.35%）	9（30.00%）
negative	25（80.65%）	21（70.00%）
Tumor stage		
T1	16（48.39%）	14（46.67%）
T2	4（12.90%）	5（16.67%）
T3	10（32.26%）	11 (36.67%）
T4	2（3.70%）	0（0.00%）
Lymph Node metastasis		
N0	7（22.58%）	9（29.03%）
Na	14（45.16%）	11（37.04%）
Nb	10（32.26%）	10（35.80%）

NC = paired tissue in the background of normal thyroid tissue.

HT = paired tissue in the background of Hashimoto thyroiditis thyroid tissue.

a represent *P* < 0.05.

### 3.2 Metabolic profile analysis of the samples

**Fig. 1:**
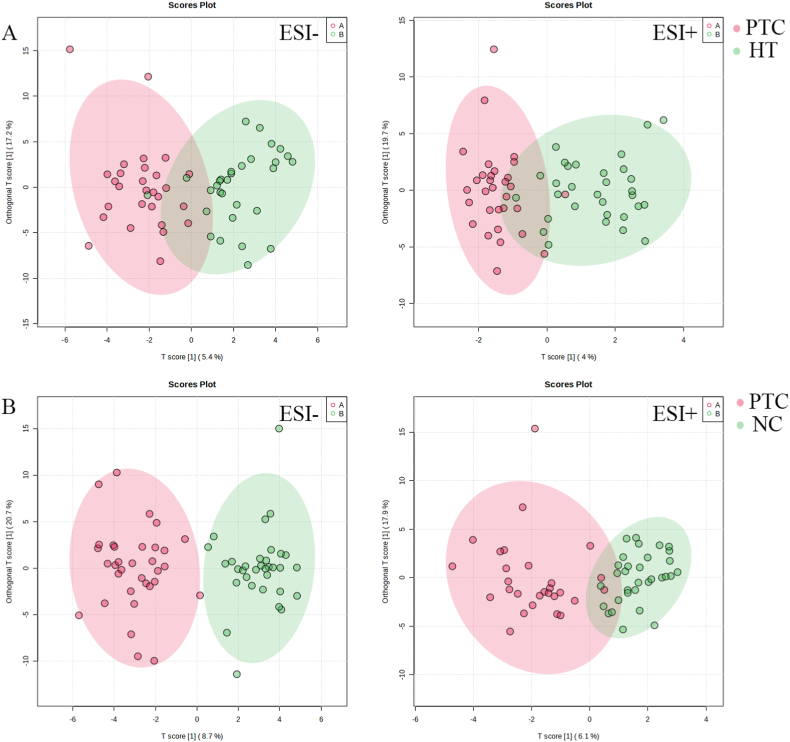
The OPLS-DA scores plot A The OPLS-DA scores plot of HT group B The OPLS-DA scores plot of NC. The red dots indicate PTC and the green dots indicate para-cancerous. (For interpretation of the references to colour in this figure legend, the reader is referred to the Web version of this article.)

**Fig. 2:**
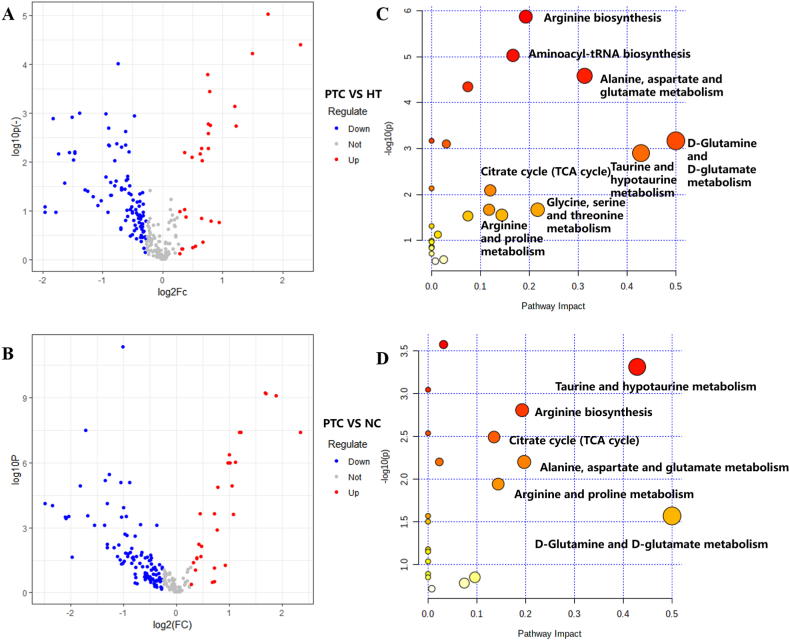
Volcano plot and Metabolic pathway of HT and NC group A The volcano plots of HT group; B The volcano plots of NC group The abscissa log2 FC in the figure indicates that the greater the point deviates from the center, the greater the difference multiple. The ordinate-log10 P indicated that if the point was closer to the top of the figure, Red indicates upregulation, blue indicated downregulation, and gray indicated no difference. C The metabolic pathway diagram of HT group; D The metabolic pathway diagram of NC group. (For interpretation of the references to colour in this figure legend, the reader is referred to the Web version of this article.)

The base peak ion (BPI) flow diagram was obtained from the data collected by UPLC-Q-TOF/MS. Data from NC and HT groups were analyzed by PCA with SIMCA-P 14.1 to evaluate the clustering characteristics of different groups. To further analyze their differences, OPLS-DA was performed. The PTC tissue was separated from adjacent tissue among the NC group or HT group, suggesting that there were some differences in metabolite profiles between the groups (Fig. 1A–B). The OPLS-DA was cross-validated by a permutation analysis (500 times). In NC group positive ion mode, the values were R2X = 0.436, R2Y = 0.794 in the OPLS-DA model, and Q2 = 0.537 in the prediction rate. In negative ion mode, the values were R2X = 0.434，R2Y = 0.792 in the OPLS-DA model, and Q2 = 0.549 in the prediction rate. In HT group positive ion mode, the values were R2X = 0.338, R2Y = 0.517 in the OPLS-DA model, and Q2 = 0.172 in the prediction rate. In negative ion mode, the values were R2X = 0.339，R2Y = 0.533 in the OPLS-DA model, and Q2 = 0.292 in the prediction rate. The results of permutation tests indicated that establishment of the OPLS-DA model was reliable without overfitting. Differential expression between NC and HT groups was visualized using volcano plots (Fig. 2A–B). There were significant differences between the two groups, and these results suggested that changes in tissue metabolic profiles can be used to distinguish patients in NC and HT groups.

### 3.3 Biomarker identification

**Fig. 3:**
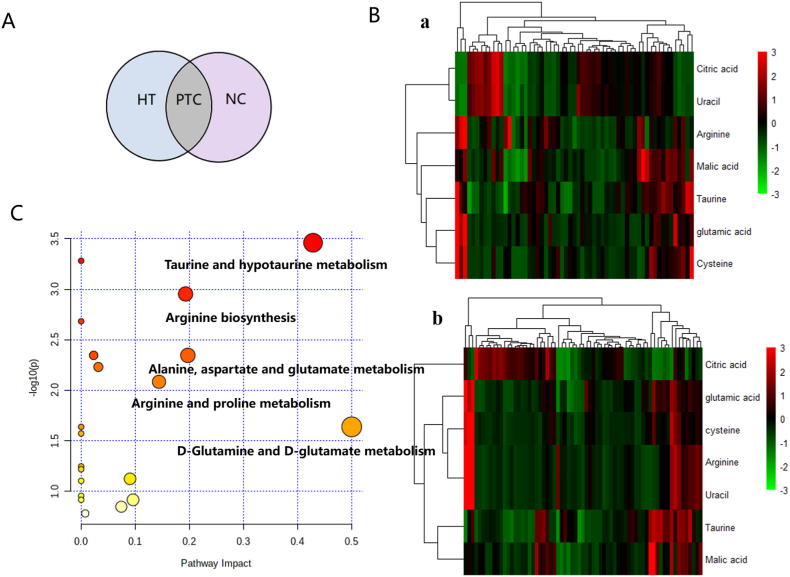
A Venn diagram; B ROC curve analyses of the ability of seven metabolites to predict PTC and adjacent tissues; C Metabolic pathway analysis of seven metabolites The horizontal coordinates in the figure indicate the impact value of the pathway and show the importance of the relevant metabolic pathway. The metabolic pathway of p ≤ 0.1 and Pathway Impact>0.1 can have enough impact between PTC tissue and its corresponding paracancerous tissue. The larger the Pathway Impact value, the greater the influencing factor.

We selected metabolites with VIP>1 (OPLS-DA) and *p* < 0.05 (*t*-test) as potential biomarkers. As shown in Fig. 3A, differential ions of HT-PTC (blue) were combined with differential ions of NC-PTC (purple) to obtain differential ions of PTC (gray). According to precise molecular weight of candidate biomarkers, the possible chemical formula was analyzed in combination with the Elemental Composition function of MassLynxV4.2 software, and biomarkers were identified by referring to the literature and HMDB, as well as by comparison with the standard substances. There were 22 differential metabolites of PTC identified in NC and HT groups, and detailed information on differential metabolites was shown in Table 2. Thirteen metabolites were noted in the HT group and ten metabolites were identified in the NC group. The biomarkers were introduced into the MetaboAnalyst 5.0 platform for visual analysis, as shown in the metabolic pathway diagram (Fig. 2C–D). Seven metabolites including arginine, glutamic acid, cysteine, citric acid, malic acid, uracil and taurine were the same among the two groups. To visualize the distribution of biomarkers in different sample groups, we used stratified cluster analysis (heatmap) to distinguish NC group and HT group. The biomarker was regarded as ordinate and the different groups were taken as the abscissa (Fig. 3B). Then, these seven biomarkers were introduced into the MetaboAnalyst 5.0 platform for visual analysis. As shown in the metabolic pathway diagram (Fig. 3C), according to impact>0.1 and raw *P* < 0.05, taurine and hypo-taurine metabolism, arginine biosynthesis, alanine, aspartate and glutamate metabolism, arginine and proline metabolism and d-glutamine and d-glutamate metabolism were obtained. The differential metabolic pathways between the two groups were aminoacyl-tRNA biosynthesis, glycine, serine and threonine metabolism.

**Table 2:** Differential metabolites in the tissue of papillary thyroid cancer patients and control subjects with positive and negative ion.

Name	Retention time (min)	ppm	Measured (*m*/*z*)	Calculated (*m*/*z*)	Formula	VIP	FC	HMDB	ESI±	Group
Serine	0.81	4.8	104.0353	104.0348	C_3_H_7_NO_3_	1.2088	0.6679	HMDB0000187	–	HT
Arginine	0.81	4.0	173.1046	173.1039	C_6_H_14_N_4_O_2_	1.4955	0.5753	HMDB0000517	±	HT/NC
Taurine	0.83	8.1	124.0078	124.0068	C_2_H_7_NO_3_S	1.2901	0.7193	HMDB0000251	–	HT/NC
Glutamine	0.83	4.8	145.062	145.0613	C_5_H_10_N_2_O_3_	1.5319	0.5895	HMDB0000641	–	HT
Malic acid	0.94	5.3	133.0144	133.0137	C_4_H_6_O_5_	1.2977	0.6754	HMDB0000744	–	HT/NC
Fumaric acid	0.94	8.7	115.0041	115.0031	C_4_H_4_O_4_	1.3843	0.6496	HMDB0000134	–	HT
Citric acid	0.98	4.7	191.0201	191.0192	C_6_H_8_O_7_	1.5497	1.6907	HMDB0000094	–	HT/NC
Uracil	0.98	9.4	111.0092	111.0195	C_4_H_4_N_2_O_2_	1.5836	1.6944	HMDB0000300	–	HT/NC
Glutamic acid	1.45	7.5	174.0415	174.0402	C_5_H_9_NO_4_	2.3958	0.2815	HMDB0000267	–	HT/NC
Isocitric acid	1.60	3.1	191.0201	191.0195	C_6_H_8_O_7_	2.0032	2.3396	HMDB0000193	–	NC
Guanine	2.28	3.3	152.0578	152.0577	C_5_H_5_N_5_O	1.6316	0.5537	HMDB0000132	+	HT
Cysteine	3.26	2.1	190.0542	190.0538	C_3_H_7_NO_2_S	1.4436	0.5505	HMDB0000574	–	HT/NC
Acetylcarnitine	4.05	3.5	202.1086	202.1079	C_9_H1_8_NO_4_	2.0275	0.3620	HMDB0000201	–	HT
Hydroxyvalproic acid	5.06	5.0	159.1029	159.1021	C_8_H_15_O_3_	1.4925	0.4257	HMDB0013900	–	NC

### 3.4 Diagnostic evaluation and pathway analysis of biomarkers

**Fig. 4:**
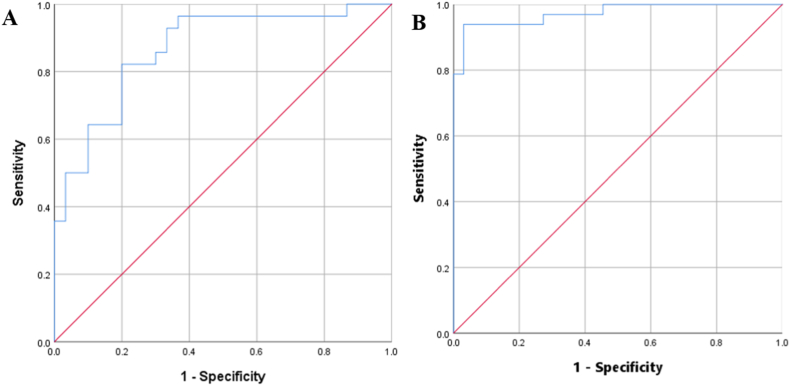
ROC curve analyses of the ability of seven metabolites to predict PTC and adjacent tissues A The AUC values of these biomarkers in HT group; B The AUC values of these biomarkers in NC group.

To assess diagnostic accuracy, we performed ROC analysis on the identified biomarkers. Table 3 showed that the AUC values of the seven biomarkers were between 0.658 and 0.787 in HT group and 0.691–0.907in NC group. We used seven biomarkers as a group of potential biomarkers of PTC, and the AUC values of these biomarkers were 0.867 in HT group (Fig. 4A) and 0.973 in NC group (Fig. 4B).

**Table 3:** Receiver operating characteristic (ROC) analysis of potential PTC biomarkers in different group.

Biomakers	AUC		95%			
	HT group	NC group	Lower		Upper	
Arginine	0.727	0.756	0.596	0.857	0.635	0.876
Citric acid	0.763	0.764	0.635	0.890	0.641	0.887
Cysteine	0.658	0.751	0.507	0.810	0.628	0.874
Taurine	0.736	0.839	0.607	0.865	0.737	0.941
Malic acid	0.717	0.691	0.576	0.859	0.554	0.827
Uracil	0.736	0.724	0.606	0.865	0.634	0.876
glutamic acid	0.787	0.907	0.665	0.909	0.839	0.975
Total	0.867	0.973	0.773	0.960	0.939	1.000

## 4 Discussion

Metabolomics is considered as an effective method to identify diagnostic biomarkers and a tool to better understand the mechanism of various diseases. A limited number of previous studies have investigated the metabolic perturbations between healthy thyroid and thyroid nodules using serum, tissue and urine samples with techniques such as gas chromatography–mass spectrometry (GC–MS) [18,21,22], liquid chromatography–mass spectrometry (LC–MS) [23], and proton nuclear magnetic resonance (1H NMR) [24]. Meantime, metabolomics has been applied in autoimmune diseases [25,26]. However, to our knowledge, this is the first report to explore the metabolic mechanisms of thyroid cancer in normal and HT backgrounds to find relevant pathways and explore their related mechanisms.

We identified the metabolic profiles of NC and HT using the UPLC-Q-TOF/MS approach, which demonstrated some similarities and differences in metabolic pathways. The link between HT and PTC has been a focus of controversy in recent years [[27], [28], [29]]. In our study, PTC tissues and adjacent tissues in HT background had specific metabolic pathways compared with normal background group, including aminoacyl-tRNA biosynthesis, glycine, serine and threonine metabolism [30]. Li et al. [31] found significant alterations in the aminoacyl-tRNA biosynthesis pathway in their metabolomic study of PTC and suggested that abnormalities in this pathway were related to abnormal protein synthesis in PTC. The contents of arginine and serine involved in the aminoacyl tRNA biosynthesis pathway may be significantly elevated in PTC tissues in the context of HT [30,32]. And, in Jiang's study [33], Kyoto Encyclopedia of Genes and Genomes Enrichment analysis showed that fatty acid degradation, Arginine, and proline metabolism have a significant impact on HT group compared to normal group.

Glycine is a simple amino acid sequence in the human body, participating in DNA methylation and the biosynthesis of glutathione and nucleotide, which also plays an important role in maintaining the antioxidant capacity and growth of cancer cells [34,35], Liu et al.’s study [26] found glycine and l-serine levels were reduced in patients with autoimmune thyroid disease. In Zhao's [36] experiment, glycine content in PTC was increased, which was consistent with the current study. It is worth noting that Sun et al. [32]indicated that the expression rate of serine/glycine metabolism-related proteins was higher in PTC cases with BRAF V600E mutation. In addition, Chen et al. [37] found that patients with lymph node metastasis from gastric cancer have increased glycine levels. Therefore, the elevation of glycine having any relationship with lymph node metastasis of PTC in the early stage needs further study. Whether the difference in prognosis of PTC in HT background and normal background is related to this metabolism remains to be further explored.

These two groups have common metabolic pathways, such as taurine and hypo-taurine metabolism, arginine biosynthesis, alanine, aspartate and glutamate metabolism, arginine and proline metabolism and d-glutamine and d-glutamate metabolism. Based on the metabolite enrichment analysis results, d-glutamine and d-glutamate metabolism was regarded as the most important factors influencing PTC development by affecting energy metabolism, which is consistent with other studies [[38], [39], [40]]. Based on the metabolic pathways of PTC in different contexts, including normal and HT backgrounds, this study reflected more comprehensively the metabolic differences between thyroid cancer and para-cancer tissues, making up for the lack of previous research. Glutamine is a compound essential for tumor growth, as a nonessential amino acid, and plays an important role directly or indirectly in tumorigenesis and cancer cell survival [41]. Torregrossa et al. [42]used 1H-HRMAS NMR spectroscopy to find metabolic changes in benign nodular goiter, malignant (PTC, FTC, and ATC) nodules, and normal thyroid tissue, and the results showed glutamate levels were increased in tumor samples. Yu et al. [43]studied four PTC cell lines (K1, IHH4, BCPAP, and TPC-1) and tissue samples using several molecular and biochemical approaches revealing that glutamate was overexpressed in cancer specimens and played a prominent role in the development and progression of PTC. Based on the results, the d-glutamine and d-glutamate pathways could be regarded as potential targets for PTC therapy. Furthermore, another previous study had reported the association between thyroid autoimmunity and PTC, and the increased serum glutamine levels might be involved [38]. All these results require further verification.

Another finding of this study is that there may be a significant increase in taurine content in PTC tissues, and the change in taurine may lead to changes in taurine and hypo-taurine metabolism pathways. Some previous metabolomic studies on thyroid nodules found that compared with benign nodules, the contents of lactic acid and taurine were increased in malignant thyroid nodules [39,42,44]. Taurine is believed to have antioxidant properties and has been implicated in the treatment of neurodegenerative disease, atherosclerosis, coronary heart disease, and prostate cancer [45,46]. It has also been found that taurine can inhibit tumor cell proliferation, enhance apoptosis, inhibit angiogenesis and play an anti-tumor role [47]. Hence, it will be essential to unravel the biological mechanism underlying taurine and hypo-taurine metabolism and other related factors in thyroid cancer.

The metabolism of PTC is complex; a single biomarker is difficult to accurately indicate thyroid cancer. In fact, we identified arginine, glutamic acid, cysteine, citric acid, malic acid, uracil and taurine as combined biomarkers for PTC diagnosis. These seven metabolites could be defined as a combinatorial biomarker to assist needle biopsy for PTC diagnosis, as demonstrated by ROC analysis (Table 3 and Fig. 4). Therefore, the potential combinatorial biomarkers provide a new direction for exploring auxiliary methods for PTC diagnosis, although a large number of samples are needed to confirm this diagnostic mode.

This study has limitations. First, we only had a small sample of patients that met our inclusion criteria. Further large-scale prospective studies are needed. Second, due to the small sample size, more detailed grouping was not possible, such as metabolic differences between different gender groups and whether there are genetic mutations. In fact, these potential metabolic biomarkers need to be validated in future multicenter, large-scale populations. Finally, in vitro and in vivo studies should be conducted to understand the underlying mechanism.

## 5 Conclusion

PTC tissues showed similar pathways such as taurine and hypo-taurine metabolism, arginine biosynthesis, alanine, aspartate and glutamate metabolism, arginine and proline metabolism and d-glutamine and d-glutamate metabolism, suggesting that tumors share common metabolic pathways resulting from the increased need for energy, macromolecular precursors, and protein and lipid synthesis. The correlation of PTC with HT may be related to aminoacyl-tRNA biosynthesis, glycine, serine and threonine metabolism.

## Ethics approval

The institutional review board and ethic committee of Tianjin Medical University General Hospital and Tianjin Medical University General Hospital Airport Site approved the ethical, methodologic, and protocol aspects of this investigation (Biomarkers in early diagnosis of thyroid follicular carcinoma based on metabonomics, IRB2021-005-01). We confirm that all methods in the current study were carried out in accordance with the relevant guidelines and regulations. Informed consent was obtained from all participants.

## Data availability statement

The datasets used and/or analyzed during the current study are available from the corresponding author on reasonable request.

## CRediT authorship contribution statement

**Danyang Sun:** Visualization, Writing – original draft, Writing – review & editing. **Yujie Zhang:** Data curation, Formal analysis. **Dan Wang:** Investigation, Methodology. **Xue Zhao:** Data curation, Investigation. **Rui Han:** Investigation, Methodology, Software. **Ning Li:** Methodology, Supervision. **Xue Li:** Data curation, Formal analysis, Project administration. **Tingwei Li:** Data curation, Methodology. **Peng Wang:** Conceptualization. **Qiang Jia:** Methodology, Supervision. **Jian Tan:** Supervision, Visualization, Writing – review & editing. **Wei Zheng:** Project administration, Visualization. **Lili Song:** Project administration, Supervision. **Zhaowei Meng:** Funding acquisition, Supervision, Visualization.

## Declaration of competing interest

The authors declare that they have no known competing financial interests or personal relationships that could have appeared to influence the work reported in this paper.

## Appendix A Supplementary data

figs1 figs1

figs2 figs2

figs3 figs3
