# Supplementary figures and images for: Experimental study on changes in metabolic mechanism of papillary thyroid carcinoma complicated with Hashimoto’s thyroiditis
Source: Heliyon. 2023 Oct 5;9(10):e20661. doi: 10.1016/j.heliyon.2023.e20661 (PMC10582305; doi:10.1016/j.heliyon.2023.e20661)

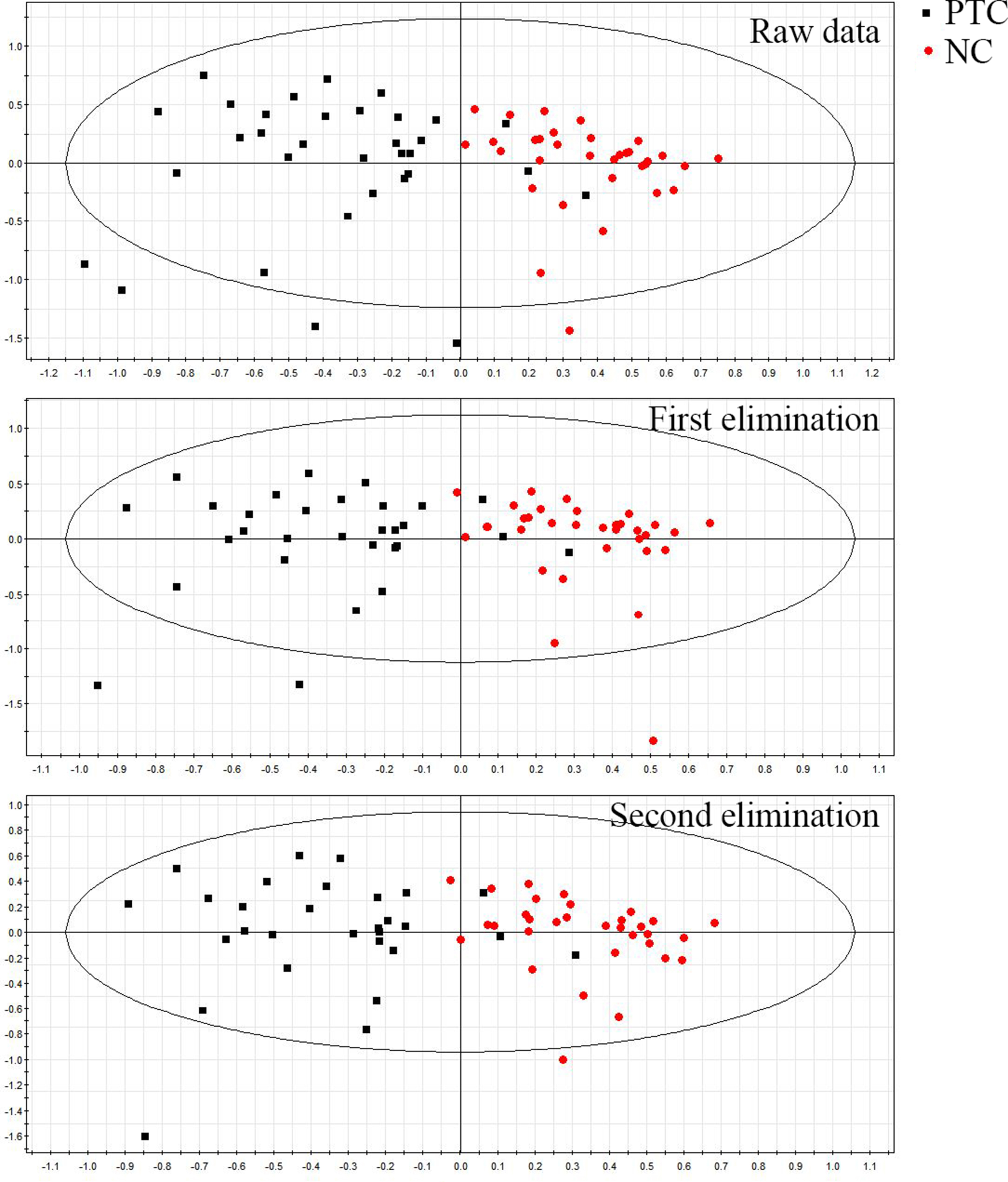

Supplement: figs1 [file mmcfigs1.jpg]

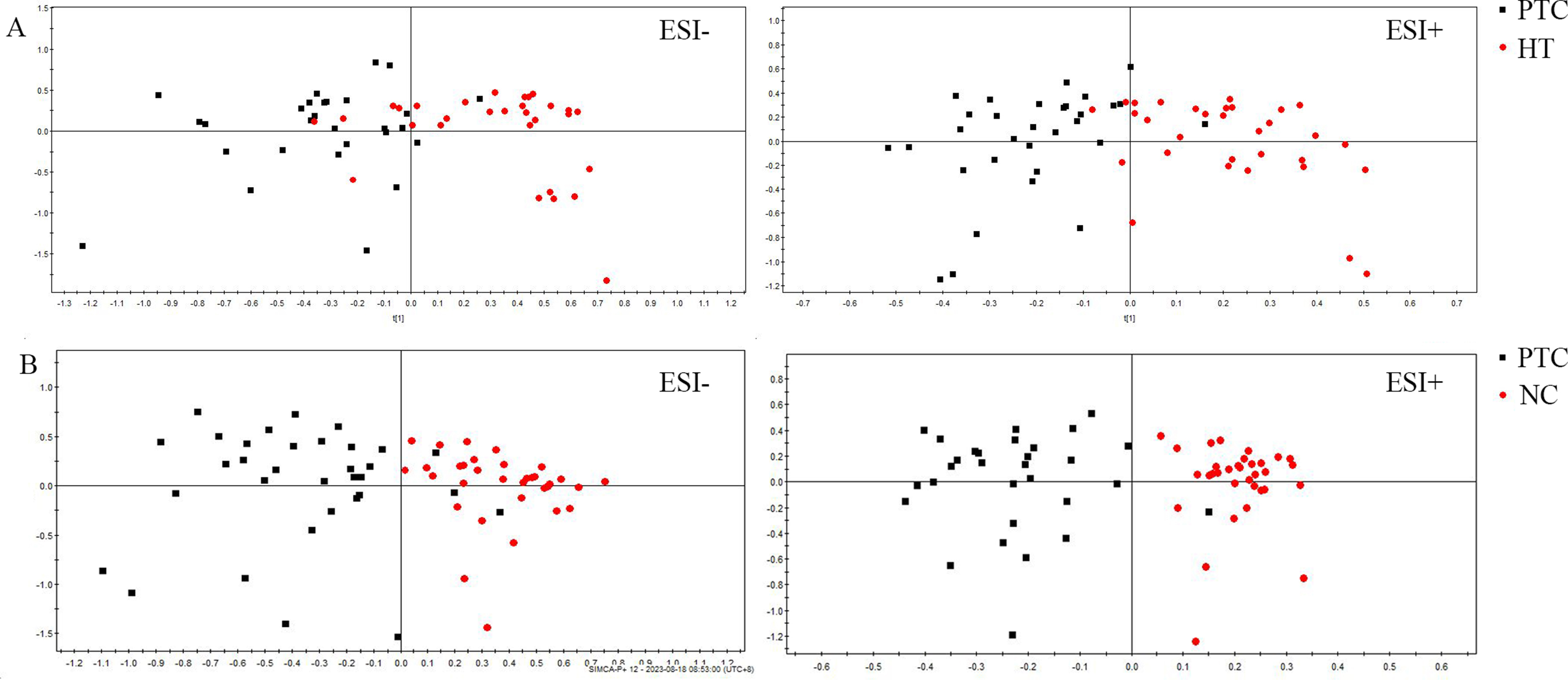

Supplement: figs2 [file mmcfigs2.jpg]

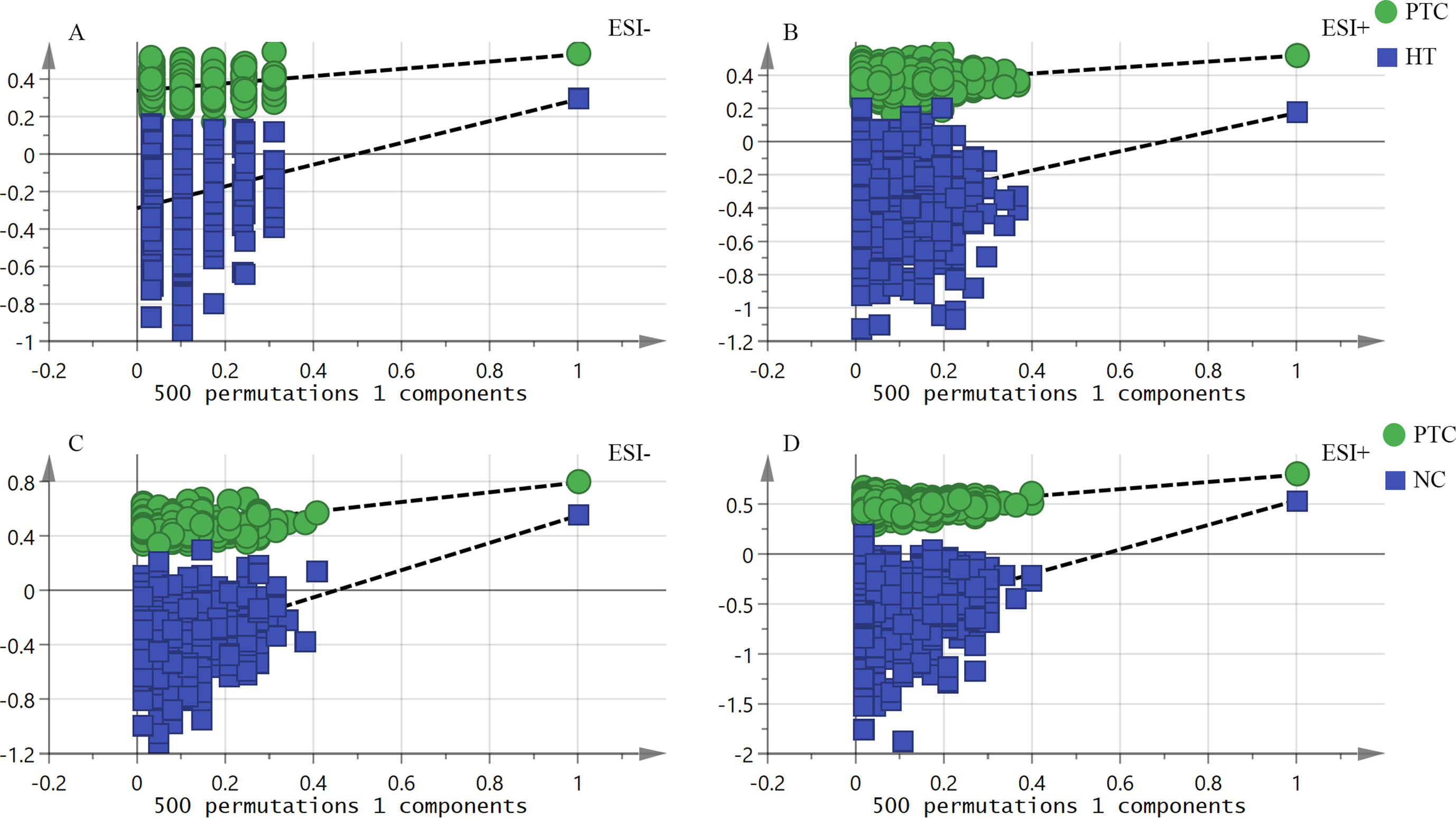

Supplement: figs3 [file mmcfigs3.jpg]
